# Seasonal changes in energy expenditure, body temperature and activity patterns in llamas (*Lama glama*)

**DOI:** 10.1038/s41598-017-07946-7

**Published:** 2017-08-08

**Authors:** Alexander Riek, Lea Brinkmann, Matthias Gauly, Jurcevic Perica, Thomas Ruf, Walter Arnold, Catherine Hambly, John R. Speakman, Martina Gerken

**Affiliations:** 1 Department of Animal Sciences, University of Göttingen, Albrecht-Thaer-Weg 3, 37075 Göttingen, Germany; 2 Faculty of Science and Technology, Free University of Bolzano, Bolzano, Italy; 3 Research Institute of Wildlife Ecology, Department of Integrative Biology and Evolution, University of Veterinary Medicine, Vienna, Austria; 4 Institute of Biological and Environmental Sciences, University of Aberdeen, Aberdeen, AB24 2TZ UK; 5 Institute of Genetics and Developmental Biology, State Key Laboratory of Molecular Developmental Biology, Chinese Academy of Sciences, 100101 Beijing, PR China

## Abstract

Mammals typically keep their body temperature (T_b_) within a narrow limit with changing environmental conditions. There are indications that some wild ungulates can exhibit certain forms of energy saving mechanisms when ambient temperatures are low and/or food is scarce. Therefore, the aim of the study was to determine if the llama, one of the most extensively kept domestic livestock species, exhibits seasonal adjustment mechanisms in terms of energy expenditure, T_b_ and locomotion. For that purpose llamas (N = 7) were kept in a temperate habitat on pasture. Locomotor activity, T_b_ (measured in the rumen) and the location of each animal were recorded continuously for one year using a telemetry system. Daily energy expenditure was measured as field metabolic rate (FMR). FMR fluctuated considerably between seasons with the lowest values found in winter (17.48 ± 3.98 MJ d^−1^, 402 kJ kg^−0.75^ d^−1^) and the highest in summer (25.87 ± 3.88 MJ d^−1^, 586 kJ kg^−0.75^ d^−1^). Llamas adjusted their energy expenditure, T_b_ and locomotor activity according to season and also time of day. Thus, llamas seem to have maintained the ability to reduce their energy expenditure and adjust their T_b_ under adverse environmental conditions as has been reported for some wild ungulates.

## Introduction

Endothermic mammals usually have to keep their body temperature (T_b_) in a narrow limit of 37–39 °C with changing ambient temperatures (T_a_)^1, 2^, which comes at a high energetic cost. Endotherms therefore are confronted with the challenge to maintain a comparatively high energy intake in the face of seasonal variation in food quantity and quality to keep the T_b_ in that aforementioned narrow limit^3^. Many small animals (<1000 g) counter this challenge by employing energy saving mechanisms such as torpor or hibernation i.e. decreasing metabolic rate and T_b_ substantially, during times of adverse environmental conditions^4–6^. With the exceptions of bears and badgers, larger animals (>5000 g) are not known to use these energy saving mechanisms. Recent studies, however, revealed that some ungulates can decrease their metabolic rate and T_b_ to some degree and thus adjust their daily energy expenditure (DEE) according to season. So far, this has been shown for the red deer (*Cervus elaphus*)^7^, Przewalski horses (*Equus przewalski*)^8^, Alpine ibex (*Capra ibex*)^9^ and Shetland ponies (*Equus caballus*)^10^. Additionally, less comprehensive studies mainly based on respirometry of captive specimens have suggested seasonal variation in the metabolism of several cervid species (reviewed in Mauget *et al*.)^11^ and other ruminants such as muskoxen (*Ovibos moschatus*)^12^ or Arabian oryx (*Oryx leucoryx*)^13^.

In its native region of South America the llama is used as a wool and meat producer, but also as a beast of burden. The llama as such is an integral part of the life of the rural population in the High Andes and contributes to a large extent to their overall income^14^. In the last two to three decades the llama also gained popularity in North America, Europe and Australia, mainly as pet animal, wool producer or for landscape management purposes^15^. However there is still a lack of scientific literature especially in the fields of nutrition and energetics^16–18^. Furthermore so far there are no studies investigating seasonal adjustment mechanisms with regard to energy expenditure and T_b_ in South American camelids. In this regard the llama is a particular well suited model animal because it is one of the most robust livestock species, which can be described as a primary population^19^.

Therefore, the aim of our study was to determine if the llama, one of the most extensively kept livestock species, exhibits seasonal and ultradian adjustment mechanisms in terms of energy expenditure, T_b_ and locomotion under Central European climatic conditions. In particular we wanted to determine if differences in energy expenditure during different seasons exist and how T_a_ is correlated with T_b_ and locomotor activity (LA).

## Results

### Climatic conditions

**Figure 1:**
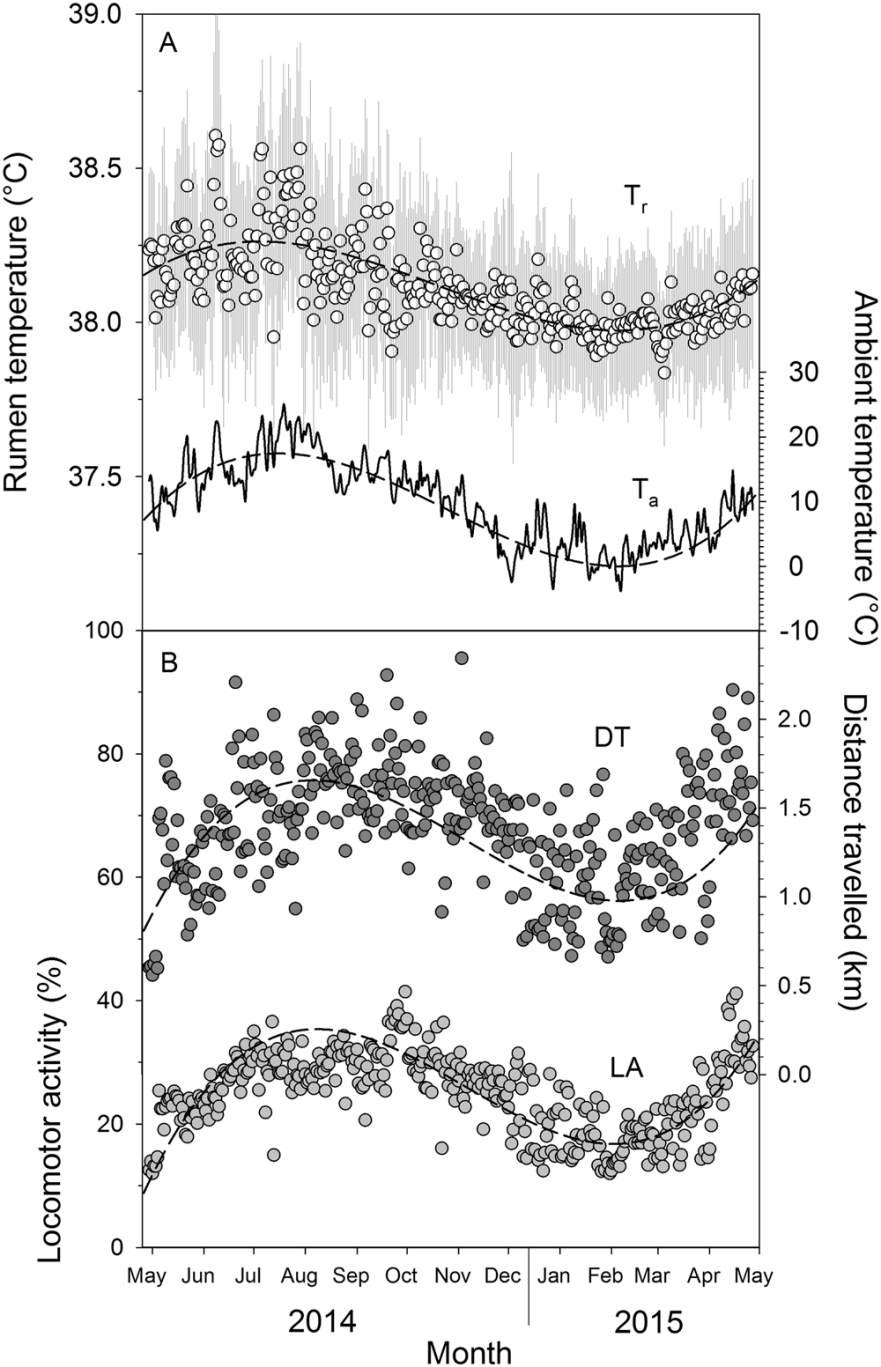
Yearly rhythm of rumen and ambient temperature as well as locomotor activity and distances travelled in llamas. Data are daily average rumen temperature (T_r_, white dots with average minima and maxima, N = 7) and daily average ambient temperature (T_a_, black solid line; **A**) as well as daily average locomotor activity (LA, light grey dots) and distances travelled (DT, dark grey dots; **B**) in adult non-pregnant llama dams during one year (29 April 2014–28 April 2015). Black dashed lines are best fit models for each parameter in A and B (see text for model equations). Values for T_r_, LA and DT are means of seven animals over a total of 2321 recording days.

The climatic conditions during the year of our study (29 Apr 2014–28 Apr 2015) were in the normal range expected for this Central European location. The T_a_ fluctuated substantially over the course of the study with daily averages ranging from 25.0 ± 9.28 °C in summer to −3.5 ± 2.1 °C in winter (Fig. 1A). The course of the average daily annual T_a_ was best described by a third degree polynomial (T_a_, °C = 4E-06x^3^ − 0.002x^2^+ 0.27x + 7.97, R² = 0.81, P < 0.001; were x is the day of study). Rainfall occurred on 157 of the 365 study days. The highest daily rainfall occurred on 29 July with 34.6 mm. The total annual rainfall over the one year study period was 867 mm. During winter there were 32 days with snow heights over 1 cm and during the entire study period there were 69 days with frost. The mean daily averages, mean daily maxima and mean daily minima for T_a_ and RH during the time of the four FMR measurements in spring, summer, autumn and winter are presented in Table 1.

**Table 1:** Ambient temperature and relative humidity during two weeks at four different seasons (spring, summer, autumn and winter, see text for details) under central European climatic conditions.

Variable		Spring	Summer	Autumn	Winter
Daily ambient temperature	(°C)	9.90 ± 2.15	17.53 ± 1.94	7.98 ± 2.00	0.73 ± 1.35
Daily minimum	(°C)	2.18 ± 2.90	11.52 ± 1.06	3.83 ± 2.86	−1.40 ± 2.41
Daily maximum	(°C)	17.52 ± 3.98	25.01 ± 4.20	13.39 ± 2.35	3.01 ± 1.87
Daily range	(°C)	15.33 ± 5.79	13.49 ± 4.48	9.56 ± 3.96	4.41 ± 2.82
Daily relative humidity	(%)	78.60 ± 9.17	81.62 ± 4.63	96.10 ± 3.74	97.28 ± 3.01
Daily minimum	(%)	48.28 ± 17.47	50.72 ± 10.97	77.80 ± 11.46	87.00 ± 8.80
Daily maximum	(%)	102.38 ± 4.08	101.22 ± 2.43	104.78 ± 1.22	103.85 ± 2.15
Daily range	(%)	54.01 ± 18.49	50.50 ± 12.59	26.98 ± 12.03	16.75 ± 9.08

Values are means ± sd.

### Body temperature

**Figure 2:**
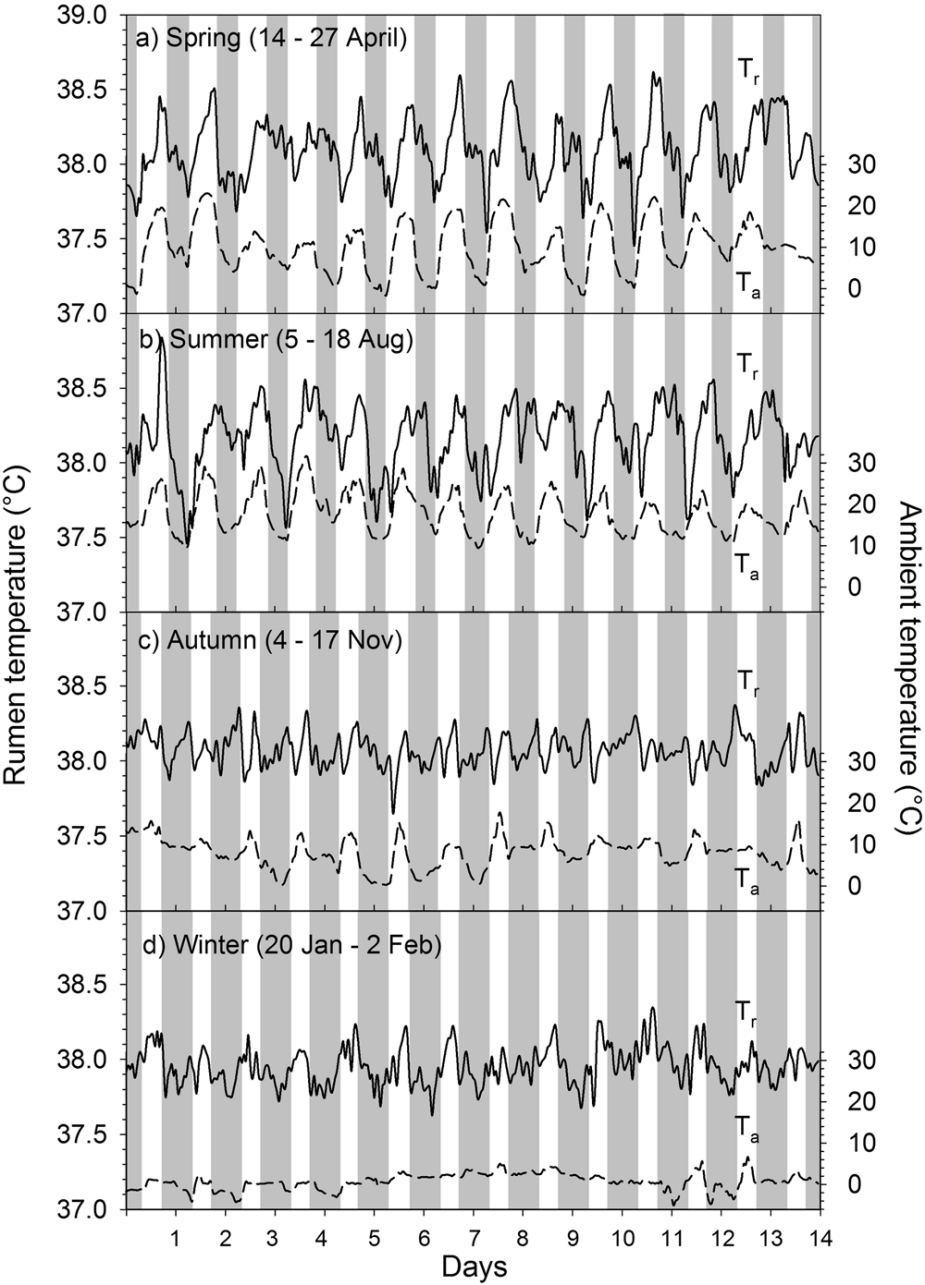
Rumen temperature in llamas. Data show the diurnal rhythm of ambient temperature (T_a_, dashed line) and average rumen temperature (T_r_, solid line) in adult non-pregnant llama dams (N = 7) during two weeks in four different seasons (spring, summer, autumn, winter) under central European climatic conditions. Grey shaded areas indicate night-phase. Axis range for T_r_ and T_a_ is the same for each season.

**Figure 3:**
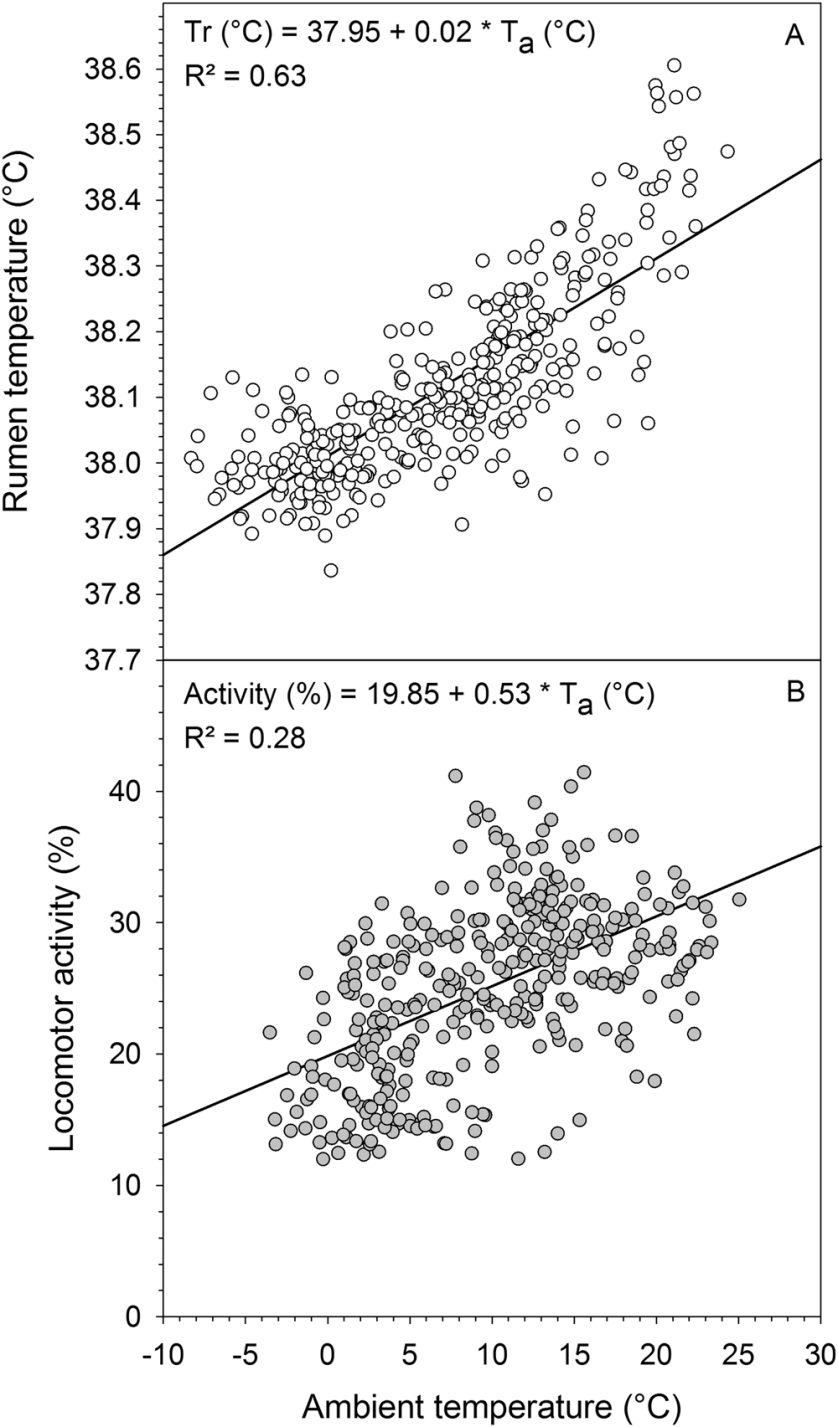
Rumen temperature and locomotor activity in llamas in relation to ambient temperature. Panels show the relationship between average daily ambient temperature (T_a_) and average daily rumen temperature (T_r_, **A**) and average daily locomotor activity (**B**) in adult non-pregnant llama dams (N = 7) during one year under Central European conditions.

The daily mean T_r_ during the entire study of one year was 38.11 ± 0.41 °C. There was a distinct fluctuation of daily mean T_r_ over the course of the study with the lowest daily mean T_r_ of 37.64 ± 0.36 °C recorded in winter and the highest of 38.86 ± 0.48 °C in summer. Similar to T_a_, the course of the average daily T_r_ over the study period was best described by a third degree polynomial (T_b_, °C = 5E-08x^3^ − 3E-05x^2^ + 0.003x + 38.18, R² = 0.54, P < 0.001; Fig. 1A, were x is the day of study). The lowest absolute T_r_ recorded was 36.02 °C and the highest was 39.98 °C. During spring and summer daily mean minimum T_r_ (T_r min_) and daily mean maximum T_r_ (T_r max_) varied considerably. Towards autumn and winter however these high variations decreased. This trend was also evident when considering T_r_ during the four FMR measurements, i.e. the daily T_r_ range (amplitude) was significantly lower in winter and autumn then during summer (Table 2). Over the entire study period of one year, daily T_r min_ was lowest in December (37.08 °C) and highest in August (37.89 °C). Similarly, lowest daily T_r max_ were recorded in March (38.27 °C) and highest in August (39.49 °C). Typically T_r max_ and T_r min_ occurred around late afternoon and early morning, respectively over the entire study including the four FMR measurement points (Fig. 2). Average daily T_r_ followed the T_a_ pattern throughout the year (Fig. 1A) and had a highly significant positive relationship with average daily T_a_ (Fig. 3A). Average daily T_r_ was also highly correlated with daily maximum (r = 0.75, P < 0.001) and minimum T_a_ (r = 0.74, P < 0.001).

**Table 2:** Average physiological and behavioural variables in llama dams (n = 7) during two weeks at four different seasons (spring, summer, autumn and winter, see text for details) under central European climatic conditions.

Variable		Spring	Summer	Autumn	Winter	SEM	F-value	Season P-value
Body mass	(kg)	150.4^d^	154.4^c^	163.7^a^	159.1^b^	9.13	36.56	<0.001
Body condition score	(points)	3.11^b^	3.14^bc^	3.38^a^	3.36^ac^	0.21	6.25	0.004
Field metabolic rate	(MJ d^−1^)	18.33^bc^	25.87^a^	20.70^abc^	17.48^c^	1.53	6.14	0.005
	(kJ kg^−0.75^ d^−1^)	401^b^	586^a^	427^b^	402^b^	37.5	5.87	0.006
Total body water	(%)	60.97	63.49	63.94	63.92	1.61	2.31	0.112
Total water intake	(L d^−1^)	6.55^b^	9.17^a^	7.55^ab^	4.22^c^	0.44	23.35	<0.001
Daily activity	(%)	32.80^a^	29.32^ab^	26.31^b^	16.08^c^	1.03	54.90	<0.001
Distance travelled	(km d^−1^)	1.64^b^	1.74^a^	1.50^c^	1.01^d^	0.03	185.6	<0.001
Daily body temperature	(°C)	38.55^ab^	38.62^a^	38.53^b^	38.40^c^	0.03	20.58	<0.001
Daily minimum	(°C)	37.49^ab^	37.44^ac^	37.60^b^	37.40^c^	0.05	17.45	<0.001
Daily maximum	(°C)	38.64^ab^	38.75^b^	38.52^a^	38.47^a^	0.06	9.39	0.001
Daily range	(°C)	1.15^ab^	1.30^a^	0.92^b^	1.06^b^	0.08	7.94	0.002

Values are LS-Means with the corresponding SEM, F- and P-value. ^a,b,c,d^Means within a row not sharing the same superscript differ by *P* < 0.05.

### Energy expenditure and water turnover

**Figure 4:**
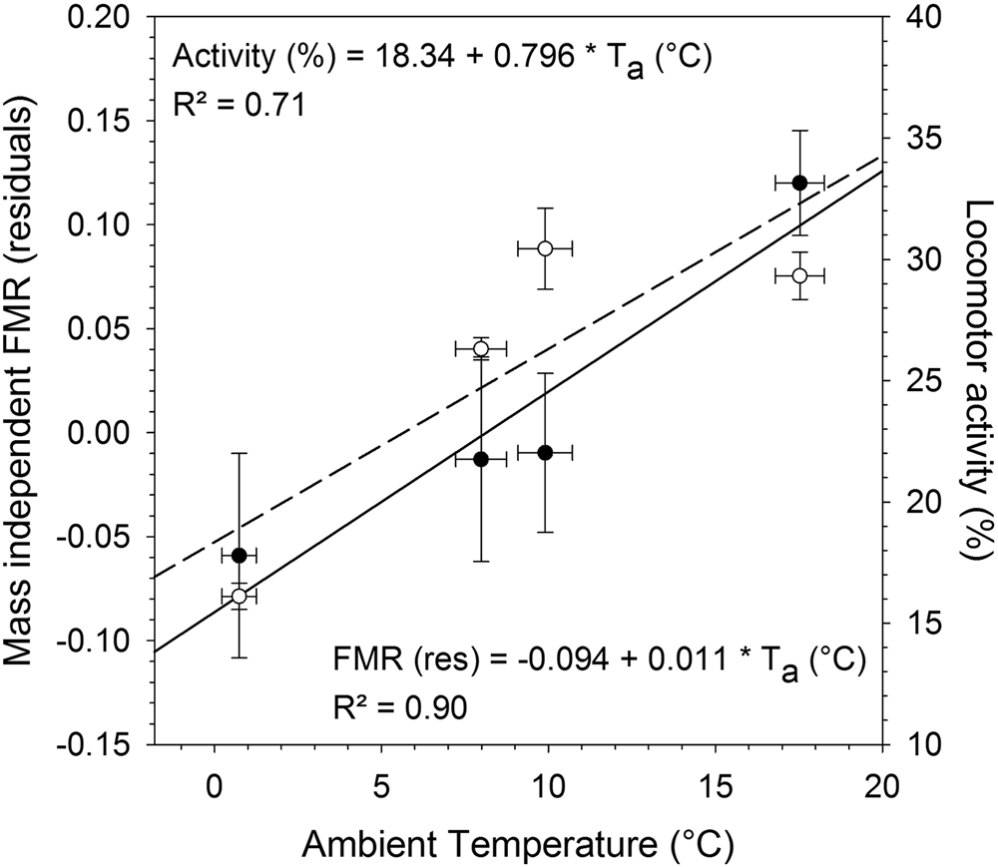
Field metabolic rate (FMR) and locomotor activity in llamas. Date are average FMR (•) and activity (○) values of llama dams (N = 7) with the corresponding fit lines (FMR: solid line; Activity: dashed line) measured at four different seasons, i.e. ambient temperature ranges. FMR values are given as residuals from the regression of FMR on body mass.

Energy expenditure in llamas measured as field metabolic rate (FMR) varied considerably between the four measurements, i.e. at different seasons (Table 2, Fig. 4). In winter, when T_a_’s were on average 0.73 ± 1.35 °C but decreased on some days to values below −6 °C, the FMR was significantly lower (17.38 ± 3.98 MJ d^−1^, 402 kJ kg^−0.75^ d^−1^) compared to values measured during summer (25.87 ± 3.88 MJ d^−1^, 586 kJ kg^−0.75^ d^−1^) when T_a_’s were on average 17.53 ± 1.94 °C but reached on some days over 30 °C. Thus DEE was about 30% lower in winter compared to summer. FMR was also higher (P < 0.001) in summer compared to spring but there were no differences (P > 0.05) between summer and autumn or winter and autumn (Table 2). Furthermore, mass independent FMR had a significant positive relationship with T_a_ (R² = 0.90, P < 0.001, Fig. 4). After the effect of body mass was removed, average residual variation of DEE explained to some degree average T_b_ (r = 0.78, P < 0.001), LA (r = 0.37, P < 0.05) and distances travelled (r = 0.63, P < 0.01).

Total body water (TBW) ranged from 57 to 69% of body mass between individual animals but average TBW did not differ between seasons (Table 2). However, total water intake (TWI) differed significantly between seasons. In summer animals ingested more than double the amount of water (9.17 ± 1.65 l d^−1^) compared to winter (4.22 ± 0.33 l d^−1^; Table 2). The TWI was also higher (P < 0.001) in summer than in spring and in spring TWI was higher than in winter. However, no differences (P > 0.05) were detected between autumn and summer or autumn and spring.

### Locomotor activity and distances covered

**Figure 5:**
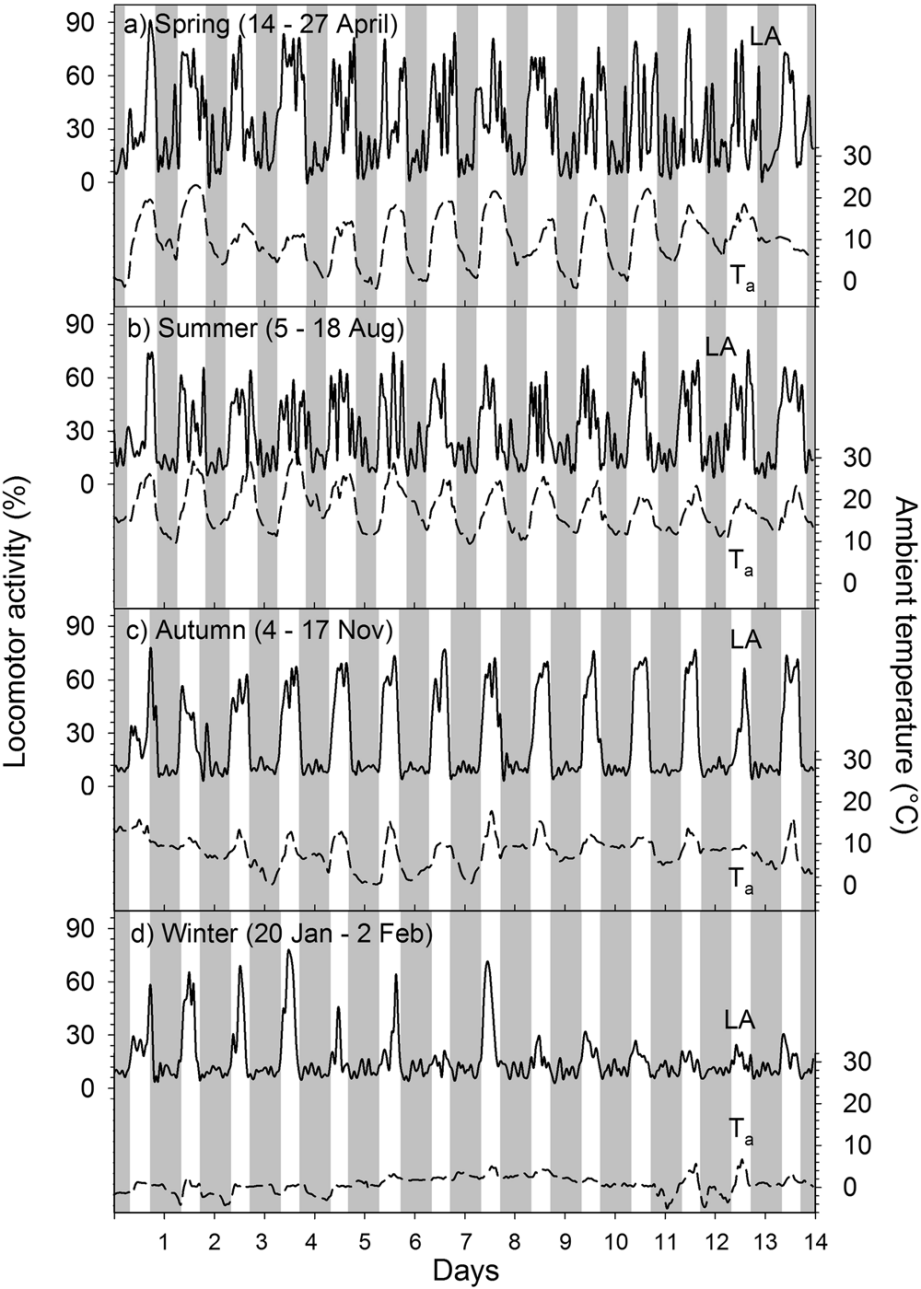
Locomotor activity in llamas. Data show the diurnal rhythm of ambient temperature (T_a_, dashed line) and mean daily locomotor activity (LA, solid line) in adult non-pregnant llama dams (N = 7) during two weeks in four different seasons (spring, summer, autumn, winter) under central European climatic conditions. Grey shaded areas indicate night-phase. Axis range for LA and T_a_ is the same for each season.

During our study mean daily LA followed a similar pattern as T_a_ (Fig. 1A) with the lowest daily average LA recorded in winter and the highest in summer (Fig. 1B). The course of average daily LA over the study period of one year was therefore, similar to T_a_, best described by a third degree polynomial (LA, % = 5E-06x^3^ − 0.003x^2^ + 0.42x + 13.48, R² = 0.56, P < 0.001, were x is the day of study). Daily average LA had thus a significant positive relationship with daily average T_a_ (Fig. 3B). This trend was also evident during the FMR measurement periods. The daily average LA in winter was with only 16.1 ± 1.4% significantly (P < 0.001) lower compared to autumn (26.3 ± 1.2%), summer (29.3 ± 2.6%) and spring (32.8 ± 4.4%, Table 2). As can be seen in Fig. 5, the diurnal LA rhythm varied between seasons, with many irregular activity peaks throughout the day in spring and summer, distinct daily peaks in autumn and infrequent activity during the measurement days in winter.

The average daily distances covered by the animals had a positive significant relationship with LA (LA, % =14.50 * Distance travelled, km + 4.94, R² = 0.58, P < 0.001; not shown). Similar to daily activity, animals covered on average significantly (P < 0.001) more distance in summer compared to winter and thus the model best describing the course of daily distances covered during the time of the study was again a third degree polynomial (Daily distances covered, km = 2E-07x^3^ − 0.0001x^2^ + 0.019x + 0.79, R² = 0.38, P < 0.001; Fig. 1B, were x is the day of study). During the FMR measurements average daily distances covered differed significantly between each season and were highest in summer and lowest in winter (Table 2). Over the entire year the average daily distances covered per day ranged from 0.66 ± 0.12 km to 2.8 ± 0.43 km.

## Discussion

Our study is the first in a camelid that combines the doubly labelled water method for measuring DEE with a telemetry system measuring locomotion and T_b_, as well as estimating the distances covered by GPS. Furthermore, our study presents the first continuous long-term T_b_ and activity measurements in a South American camelid. The data show that llamas seem to have maintained the ability to reduce their DEE and activity and to a certain degree adjust their T_b_ according to season and time of the day as has been reported for some wild ungulates.

Some studies on wild and domestic ungulates have reported substantial reductions in DEE during adverse environmental conditions^7, 9, 10, 20^. In our study on llamas, we found similar adaptations in DEE. The DEE of the animals in our study varied considerably between seasons with FMR values in summer being on average 30% higher compared to winter (Table 2, Fig. 4). This can be to some degree explained by the higher activity levels during summer (Table 2, Fig. 5) when animals were grazing and also by a higher food intake. For example studies on alpacas^21^ and cervids^7, 22–24^ have shown that the seasonality of metabolism is linked to the level of food intake.

The T_a_ fluctuations in our study with an average daily maximum of 25.0 ± 4.2 °C in summer and a daily minimum of −1.4 ± 2.41 °C in winter are typical for temperate regions like our Central European study site (Table 2). Compared to the High Andes of South America, the native region of the llama, where T_a_ can fluctuate by 40–50 °C per day, the seasonal and daily T_a_ fluctuations in our study can be considered as moderate. Nevertheless, during lower T_a_’s, e.g. in winter, thermoregulatory costs for endothermic animals usually increase to keep the T_b_ within a narrow limit^1, 2^ and thus resulting in an increased DEE^25^. In our study however we observed the opposite, i.e. decreased DEE in winter when T_a_’s were low and increased DEE in summer when T_a_’s were high. The thermal neutral zone, which is the range of T_a_ in which T_b_ in an animal is only regulated by the control of sensible heat loss and thus does not require additional energy for thermoregulation^26, 27^, has so far not been directly measured in the llama. However data from guanacos (*Lama guanicoe*) which is the wild ancestor of the llama, suggest that the breadth of the thermal neutral zone seems to be quite large with a lower critical temperature being in the range of −10 to −15 °C and an upper critical temperature of about 20 °C^27, 28^. This large thermal neutral zone is most likely due to the ability of guanacos and llamas to use peripheral vasoconstriction and local heterothermy. Furthermore guanacos and llamas employ behavioural adjustments to minimise heat loss during cold exposure^29^. In endothermic animals the energetic costs for thermoregulation increase when T_a_ decreases below the lower critical temperature or increase above the upper critical temperature, thus defining the thermal neutral zone of an animal^1^. It can be assumed that our lamas were within their thermal neutral zone during the cooler seasons, i.e. winter, autumn and spring when T_a_ did not exceed 20 °C or fell below −10 to −15 °C (Table 1). Thus suggesting that no additional energy was needed for thermoregulation during these seasons since the decrease of T_r_ during winter shifted the zone of thermoneutrality to a lower temperature indicating that our animals were under conditions of thermoneutrality for most of the winter. During the summer months however when T_a_ exceeded 20 °C, the DEE was significantly higher compared to winter or spring. Furthermore LA also differed between summer and winter. Therefore the lower DEE measured in winter compared to summer is most likely due to a reduction in LA and T_b_, evidenced by a positive and significant relationship between FMR and LA as well as T_b_. Furthermore animals were within their thermal neutral zone and thus did not need the extra cost for thermoregulation. Conversely, the increased DEE in summer can be explained by an increased LA and an increased cost for thermoregulation because the T_a_ most likely exceeded the upper critical temperature at least on some days during the summer measurement. Similar results have been also found for red deer^7^, Przewalski horses^8^ and ibexes^9^ indicating that during low T_a_ animals reduce their activity and thus lower their energy expenditure. This has been also observed in smaller mammals such as red squirrels (*Tamiasciurus hudsonicus*)^30^ and least weasels (*Mustela nivalis*)^31^ living in temperate or arctic regions. Interestingly other studies of animals living in warmer regions such as kangaroo rats (*Dipodomys merriami*) or white footed mice (*Peromyscus leucopus*) do not show this kind of adaptation^25, 32–34^.

**Figure 6:**
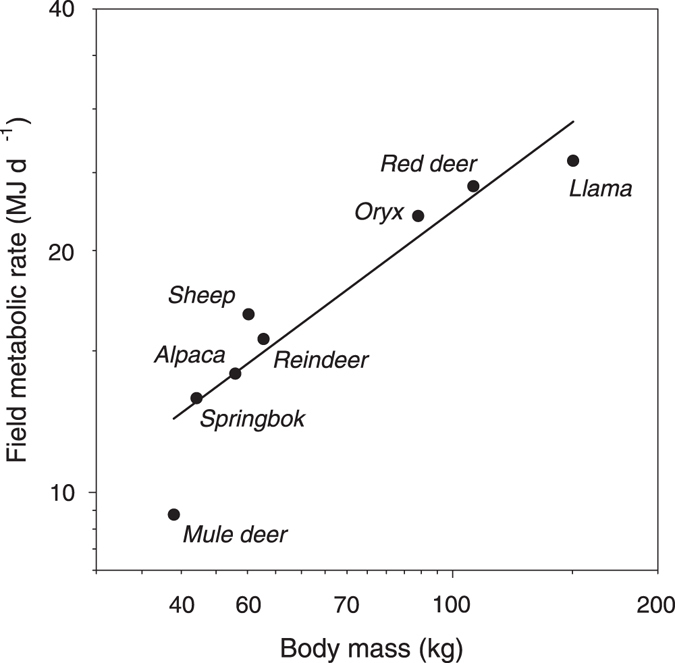
Relationship between daily energy expenditure and body mass in artiodactyls. Data are measurements of field metabolic rate (FMR) using the doubly labelled water method and body mass (BM) for eight artiodactyls (including data on llamas from the present study). The phylogenetically controlled allometric relationship is FMR (MJ d^−1^) = 1.23 BM^0.63±0.12^ (*F*_*1,7*_ = 26.2, *P* < 0.001, *R²* = 0.81, ML *λ* = 0.64) (see text for details).

Very few studies on artiodactyls are available that measured DEE using the doubly labelled water method. So far DEE has been measured in eight artiodactyls (Mule deer, *Odocoileus hemionus* ^35^; reindeer, *Rangifer tarandus* ^36^; springbok, *Antidorcas marsupialis* ^37^; red deer, *Cervus elaphus* ^38^; Arabian oryx, *Oryx leucoryx* ^13^; sheep, *Ovis aries* ^39^; alpacas, *Lama pacos* ^40^). Comparing average DEE between these species, llamas have a DEE of 28.9 MJ/d predicted from a phylogenetic corrected regression equation for artiodactyls (FMR, MJ d^−1^ = 1.23 BM^0.63±0.12^, Fig. 6). This is about 10% higher than the actual DEE measured during summer (25.9 MJ/d), which was the highest average DEE measured during the four measurement points of the present study and more than 30% higher than the value measured in winter (17.48 MJ/d) which was the lowest average DEE measured during our study. This suggests that llamas seem to have on average a lower DEE relative to other artiodactyls (with the exception of the mule deer). Similar suggestions have been also made for other camelids^18^. Interestingly, the exponent of the phylogenetic corrected regression equation for the relationship between FMR and body mass in artiodactyls (0.63, 95% CI 0.32–0.91) is very close to the one found for Metatheria (0.60)^41^. However these results need to be treated with caution as only very few data from artiodactyls are available and thus no definite conclusions can be reached or valid comparisons be made until further systematic studies on other artiodactyl species are available. Furthermore, predicting values for missing species that have not been measured from phylogenetic regression equations, as it is sometimes done in comparative analysis, are likely to be incorrect as the fit-lines are phylogenetically controlled and thus will not account for the phylogenetic history of the missing species.

The TWI calculated from isotope turnover rates differed between seasons (Table 2). During summer animals ingested with 9.17 l/d on average more than double the amount of water compared to winter (4.22 l/d). Most likely, these differences can be attributed to an increased drinking water ingestion during summer when animals displayed higher physical activities and the average daily maximum T_a_ was above 25 °C (Table 1). Furthermore animals consumed more pasture in summer, containing a higher percentage of water compared to hay, which was the main food source in winter. As explained before, when T_a_ increases above the upper critical temperature it triggers energy dependent thermoregulative mechanisms for dissipation of metabolic heat and thus keeping T_b_ within a narrow limit^42^. Therefore, substantial amounts of water will be dissipated with increasing T_a_ especially via the thermal windows at the ventral regions of the body in llamas, resulting in increased water ingestion and water turnover^43^. Additionally, the increased DEE of the animals during the summer months would have resulted in a greater production of metabolic water. These changes in water turnover between seasons however did not affect the TBW. The average TBW of 63.1% of body mass of all animals did not differ between seasons (Table 2) and was in the range of reported TBW values for ungulates (for comparison see Table 2 in ref. 10).

The daily T_b_ fluctuations, i.e. T_b_ decreasing during the night and increasing during the day were more pronounced in summer and spring when T_a_ fluctuations were higher, than in winter and autumn when T_a_ fluctuations were lower (Fig. 1A, Table 2) suggesting that animals were following a shallow daily hypometabolism. This adaptive mechanism to save energy by reducing the metabolic rate has been reported for many smaller animals (body mass < 5 kg) during the 24 h rhythm of rest and activity^6, 44^. For humans and larger animals similar results have been found^7–9, 45^. In our study average daily T_b_ amplitudes during the FMR measurement points were highest in summer (1.3 °C) and lowest in autumn (0.92 °C, Table 2). Similar average T_b_ amplitudes have been reported for alpacas (1.5 °C)^46^, angora goats (1.4 °C)^47^, blesbok (1.4 °C)^46^, pronghorn (1.0 °C)^48^ and impala (1.1 °C)^49^. However our results are daily averages of seven animals over two weeks. Looking at the individual daily T_b_ variations over the study year the highest amplitudes found ranged from 1.8 to 3.2 °C and occurred all in summer (Fig. 1). Higher T_b_ amplitudes of 6–7 °C have been reported for springboks^50^ and camels (*Camelus dromedarius*)^51^. Nevertheless the T_b_ amplitudes of our animals were higher than the normal circadian variations in T_b_ for llamas (37.5–38.6 °C)^52^ possibly using adaptive heterothermy to some degree to reduce DEE. Furthermore in our study daily T_b_ variations could be observed over the entire study period following the daily photoperiod as has also been reported for horses^8, 45, 53^, red deer^7^ and ibex^9^. Usually the 24 h T_b_ rhythm is as such an endogenous rhythm and is synchronized by the external ‘Zeitgeber’ independent of exogenous factors. This could explain why on some days T_b_ decreased at night when T_a_ did not but instead stayed rather constant, e.g. during some winter days (Fig. 2d). However, T_b_ was correlated with T_a_ throughout the study (Fig. 3A) and thus following the general rhythm of the daily T_a_ cycle. Nevertheless animals sharply increased their activity in the morning possibly resulting in an increase of T_b_. During the lower T_b_ variations in winter and autumn, i.e. when T_a_ was generally low, animals might have shifted from a short daily hypometabolism in spring and summer to a more intense hypometabolism to save energy, as has been recently shown for Shetland ponies^10^. Thus the decreased T_b_ amplitude during the cooler seasons could be explained by an adaptation to save energy. Similar observations have been recently described in grey kangaroos^54^ and the oryx^55^. Furthermore, the lowering of the T_b_ during night hours might reduce the energetic cost for thermoregulation by increasing the capacity to store heat during hot days^3, 50^. This adaptive heterothermy has been already demonstrated in a variety of other ungulates such as the eland^56^, oryx^42, 55^, giraffe^57^, Arabian sand gazelle^58^ and Thomson’s as well as Grant’s gazelles^59^. However other studies challenge these findings^60, 61^ or suggest that heterothermy in large mammals could be a sub-optimal response to environmental challenges^46^. Nevertheless in general llamas seem to lower their T_b_ under energetic constraints which must involve some trade-offs that are less energy demanding than keeping the T_b_ constant^62, 63^.

In conclusion we show that llamas, one of the most extensively kept livestock species, are able to reduce their energy expenditure under adverse environmental conditions by reducing their activity and adjusting their daily T_b_ variation. Thus llamas show distinct seasonal acclimatization similar to wild ungulates.

## Methods

### Animals and study site

The study was conducted at the research farm Relliehausen (41°46′ N, 9°41′ E) of the Department of Animal Sciences at the University of Göttingen (Göttingen, Germany) and involved seven non pregnant llama dams (age: 3–13 years, body mass: 113–174 kg). The measurements were carried out continuously for one year (29 Apr 2014–28 Apr 2015). Animals were kept on a pasture (3 ha) within a herd of 15 llama dams. All llamas had free access to a barn providing shelter from wind and rain. On pasture food consisted of natural vegetation and a mineral supplement provided by a salt lick (Eggersmann Mineral Leckstein, Heinrich Eggersmann GmbH & Co KG, Rinteln, Germany). Hay and water was available *ad libitum* for all animals throughout the experiment.

### Measurements

#### Climate

The T_a_ (resolution: 0.0625 °C) and relative humidity (RH, resolution: 0.04%) were recorded continuously throughout the year with miniature data loggers at hourly intervals (i-Button, DS1923#F5, Maxim Integrated Products, Sunnyvale, CA, USA). Precipitation data were obtained from a nearby weather station at approx. 2 km distance to the farm.

#### Telemetry and body condition score

We equipped 7 animals with a telemetry system (GPS Plus-3 Store on Board collar, Vectronic Aerospace GmbH, Berlin, Germany) described in detail elsewhere^64^. In brief, the telemetry system consists of two units, a ruminal unit (22 × 80 mm, 100 g) and a collar unit (450 g). The ruminal unit was administered *perorally* after animals were immobilized with an anaesthetic drug (Xylacin, Rompun®; Bayer HealthCare, Leverkusen, Germany, 1 ml/100 kg body mass). The ruminal unit measured T_r_ every 3 min, which was transmitted via short-distance UHF link to a data logging system located in the collar unit^9^. Furthermore, locomotor activity (LA) was also recorded every 3 min with two different activity sensors. All data were recorded every 3 min for one year and stored in the collar unit and downloaded via a laptop. Additionally the position of each animal was recorded every 30 min using a GPS device located on the back of the collar (GPS Plus-3 Store on Board collar, Vectronic Aerospace GmbH, Berlin, Germany). The body condition score (BCS), a palpable and visual assessment of the degree of fatness (BCS scale: 0 = emaciated, 5 = obese), of individual animals was recorded during the four FMR measurement times according to the system described elsewhere^65^.

#### Field metabolic rate

The FMR, TBW and TWI were determined during two weeks in the European summer (5–18 Aug 2014), autumn (4–17 Nov 2014), winter (20 Jan – 2 Feb 2015) and spring (14–27 April 2015), for each animal using the doubly labelled water (DLW) method^66, 67^. At the beginning and at the end of the FMR measurements, body mass was recorded for each llama using a mobile scale (Weighing System MP 800, resolution: 0.1 kg, Patura KG, Laudenbach, Germany) and a blood sample of 5 ml was drawn from the *Vena jugularis* of every animal to estimate the background isotopic enrichment of ^2^H and ^18^O in the body fluids (method D^68^). After taking the background sample, each llama was injected intravenously with approximately 0.16 g of DLW per kg body mass, (65% ^18^O and 35% ^2^H). The individual dose of each llama was determined prior to the injection according to its body mass. The actual dose given was gravimetrically measured by weighing the syringe before and after administration to the nearest 0.001 g (Sartorius model CW3P1–150IG-1, Sartorius AG, Göttingen, Germany). The llamas were then held in the stable with no access to food or water for an 8-h equilibration period, after which a further 5 ml blood sample was taken. After dosing, additional blood samples were taken at 7 and 14 days to estimate the isotope elimination rates.

All blood samples were drawn into blood tubes containing sodium citrate. Whole blood samples were pipetted into 0.7 ml glass vials and stored at 5 °C until determination of ^18^O and ^2^H enrichment. Blood samples were vacuum distilled^69^, and water from the resulting distillate was used to produce CO_2_^70^ and H_2_^71^. The isotope ratios^18^O: ^16^O and^2^H: ^1^H were analysed using gas source isotope ratio mass spectrometry (Isochrom μG and Isoprime respectively, Micromass Ltd, Manchester, UK). Samples were run alongside five lab standards for each isotope (calibrated to the IAEA International standards: SMOW and SLAP) to correct delta values to ppm. Isotope enrichments were converted to values of CO_2_ production using a two pool model as recommended for this size of animal^72^. We chose the assumption of a fixed evaporation of 25% of the water flux, since this has been shown to minimize error in a range of applications^73, 74^. Specifically carbon dioxide production rate (r_CO2_) per day in mols was calculated using equation A6 from Schoeller *et al*.^75^. The daily amount of energy expended measured as FMR was calculated from carbon dioxide production by assuming a respiration quotient of 0.85. Total body water (mols) was calculated using the intercept method^67^ from the dilution spaces of both oxygen and hydrogen under the assumption that the hydrogen space overestimates TBW by 4% and the oxygen-18 space overestimates it by 1%^75^. The TWI (l/day) that consists of drinking water, preformed water ingested in food and metabolic water was estimated as the product of the deuterium space and the deuterium turnover rate^76^.

### Analysis

The measurements of T_r_ had non-physiological declines that could be attributed to the ingestions of water and cold food^64^. These data points were removed by visually checking the raw data. In this cleaned data set, T_r_ values ranged from 36.02 to 39.98 °C. In total 2321 individual days were available for data analysis of LA and T_r_. Hourly and daily means were calculated using R 3.3.2^77^.

In order to compare FMR values with measured physiological variables during the time of FMR measurements a mixed model was used with animal as a random factor and season (i.e. FMR measurement periods) as a fixed factor to compare animals between seasons using the MIXED procedure in SAS version 9.2 (SAS, Inst. Inc., Cary, NC). The model residuals were normally distributed. Data are expressed as LS-Means or means ± S.D where appropriate. Furthermore FMR values were also expressed as mass independent FMR by calculating the residuals of the regression of FMR on body mass. Daily distances between continuous GPS locations for each animal were calculated with the program package ‘geosphere’^78^ in R^77^. Furthermore Spearman correlations were calculated between different variables.

To test for the generality of the relation between body mass and FMR in artiodactyls, published data^35–40^ and our results, using the summer measurements, were assessed using the phlyogenetic generalized least squares approach (PGLS)^79, 80^ in order to account for the potential lack of independence between species, because of their shared evolutionary history^81, 82^. The statistical procedure has been described in detail elsewhere^83^. In brief, the phylogeny was derived from a published mammalian supertree which includes 4510 species with updated branch lengths derived from dated estimates of divergence times^84^. The supertree for mammals was pruned to include only the species of concern, i.e. artiodactyls (*n* = 8), using the ‘Analysis in phylogenetics and evolution’ (APE) package^85^ and the ‘Analysis of evolutionary diversification’ (GEIGER) package^86^ in R^77^. The method of PGLS was implemented for the trait data using the ‘Comparative analyses of phylogenetics and evolution’ (CAPER) package^87^ in R^77^. PGLS analysis allows more flexibility than ordinary least square or independent contrasts methods through the use of a parameter (lambda, λ). The parameter λ is determined by maximum likelihood (ML) and can range between 0 (no phylogenetic signal, similar to ordinary least squares analysis) and 1 (pattern of trait data variation is fully explained by the phylogeny) and thus indicates how strong the phylogenetic signal for a certain trait or the relationship between two traits is. Intermediate values of λ indicate that the trait evolution is phylogenetically correlated, but does not follow fully a Brownian motion model^88^. A more in depth description and further mathematical details on PGLS analysis can be found in detail elsewhere^79, 89, 90^.

### Ethics

Procedures performed in our study were in accordance with the German animal ﻿ethics regulations and approved by the State Office of Lower Saxony for Consumer Protection and Food Safety (Ref. No.: 33.4-42502-05-13A393).

### Data availability

The data analysed during the current study are available from the corresponding author on reasonable request.
